# Protective Role of the Sodium Taurocholate Cotransporting Polypeptide S267F Variant Against Hepatitis B Virus Infection and Cirrhosis in the Vietnamese Population

**DOI:** 10.1002/jmv.70678

**Published:** 2025-10-28

**Authors:** Pham Xuan Huy, Le Chi Cao, Dao Thi Huyen, Tran Thi Thu Hien, Truong Nhat My, Tran Thi Thanh Huyen, Le Huu Song, Thirumalaisamy P. Velavan, Nguyen Linh Toan

**Affiliations:** ^1^ Vietnam Military Medical University Hanoi Vietnam; ^2^ Institute of Tropical Medicine University of Tübingen, and German Center for Infection Research (DZIF) Tübingen Germany; ^3^ Hue University of Medicine and Pharmacy (HUMP), Hue University Hue Vietnam; ^4^ Vietnamese German Center for Medical Research (VG‐CARE) Hanoi Vietnam; ^5^ 108 Military Central Hospital Hanoi Vietnam; ^6^ Faculty of Medicine Duy Tan University Danang Vietnam

**Keywords:** HBV genotype, hepatitis B virus (HBV), hepatocellular carcinoma, liver cirrhosis, NTCP S267F variant

## Abstract

The sodium taurocholate cotransporting polypeptide (NTCP) serves as both a hepatic bile acid transporter and an essential entry receptor for hepatitis B virus (HBV). The S267F (rs2296651) polymorphism in the NTCP gene has been associated with reduced HBV susceptibility in East Asian populations. However, its clinical relevance in the Vietnamese population remains underexplored. We investigated the association between the NTCP S267F variant and clinical outcomes in 743 Vietnamese individuals, including 429 HBV‐infected patients and 314 healthy controls (HCs). HBV genotyping, NTCP variant screening, and analysis of demographic and clinical parameters were conducted. Associations between genotypes, liver disease severity, and viral genotypes were assessed using logistic regression and non‐parametric tests. The heterozygous CT genotype of S267F was significantly less frequent in HBV patients (12%) compared to HCs (16%) (adjusted OR: 0.5; *p* = 0.013), suggesting a protective role against HBV infection. This effect was most pronounced in patients with chronic hepatitis B (CHB, OR: 0.3; *p* = 0.013) and liver cirrhosis (LC, OR: 0.4; *p* = 0.041), indicating a twofold to threefold reduced risk. No significant association was observed between the variant and hepatocellular carcinoma (HCC). HBV genotype C was associated with a significantly increased risk of progression to LC (OR: 2.4; *p* = 0.015) and HCC (OR: 2.5; *p* = 0.039) compared to predominant genotype B. The protective effect of the S267F variant was independent of HBV genotype. The NTCP S267F variant is associated with reduced susceptibility to HBV infection and progression to cirrhosis in the Vietnamese population but does not confer protection against HCC. These findings highlight the potential of host genetic factors in influencing HBV disease outcomes and may support future strategies for individualized risk assessment and management.

## 1 Introduction

Hepatitis B virus (HBV) infection remains a significant global health concern, affecting almost 300 million people worldwide [1]. It is a leading cause of liver‐related morbidity and mortality, contributing to liver cirrhosis (LC) and hepatocellular carcinoma (HCC), which are among the leading causes of cancer‐related deaths globally [2]. In Vietnam, the burden of HCC is particularly high, ranking third in liver cancer incidence and mortality rates in Southeast Asia, behind Laos and Cambodia (GLOBOCAN 2022) [3]. In 2022, Vietnam reported approximately 24,502 new cases of HCC, resulting in 23,333 related deaths, making HCC the second most common cancer and the leading cause of cancer‐related deaths in the country [3].

Molecular epidemiological studies have demonstrated significant geographic variation in the distribution of HBV genotypes, which can affect the clinical course, therapeutic response, and long‐term outcomes of infection [4]. To‐date, ten HBV genotypes (A–J) have been identified, with various subgenotypes reported. Among them, genotypes C and D are associated with a higher risk of LC and HCC than other genotypes [5]. In Vietnam, chronic HBV patients predominantly carry genotype B, followed by genotype C. Other genotypes have been reported in small numbers [6], but data on the distribution of these genotypes in patients with advanced liver disease, including LC and HCC, remain limited [7].

The sodium taurocholate co‐transporting polypeptide (NTCP) receptor, encoded by the *SLC10A1* gene, plays a critical role in HBV and hepatitis D virus (HDV) entry into hepatocytes [8]. NTCP is a transmembrane protein predominantly expressed at the basolateral membrane of liver cells, facilitating the sodium‐dependent uptake of bile acids. The N‐terminus of the pre‐S1 domain of the large HBV envelope protein specifically binds to NTCP, enabling viral entry into hepatocytes [8]. The S267F variant (rs2296651, c.800 C > T) of the *SLC10A1* gene has gained significant attention due to its protective effect against HBV infection, which may reduce the risk of progressing to severe liver disease, such as LC and/or HCC [9, 10]. This variant alters the NTCP structure, decreasing its ability to bind HBV, thereby limiting viral entry and subsequent infection. However, some studies suggest that the S267F variant may not be significantly associated with HBV complications [11]. Interestingly, the S267F variant is notably prevalent in Vietnam, occurring at a frequency of 16%, compared to 3% to 9% in other Asian populations [10, 12]. This high prevalence underscores the importance of further research to elucidate the role of this variant in HBV‐related liver disease progression in Vietnamese population.

This study aims to examine the association between the NTCP S267F variant and the risk of HBV infection progression in Vietnam, focusing on its potential role in liver disease development, including cirrhosis and HCC. Additionally, we explore the distribution and clinical impact of HBV genotypes in patients with advanced liver disease in the Vietnamese population.

## 2 Materials and Methods

### 2.1 Ethic Statement

This study was approved by the Research Ethics Committee of the Vietnam Military Medical Academy (VMMA) in Vietnam, under reference number 64/KHQS dated 7 February 2023. Written informed consent was obtained from all participants after they were provided with a detailed explanation of the study at the time of blood sampling. All experiments adhered to applicable ethical guidelines and regulations.

### 2.2 Study Cohort

A total of 314 healthy controls (HCs) and 429 HBV‐positive patients were recruited from the 103 Military Hospital and the 108 Military Central Hospital in Hanoi, Vietnam, between 2023 and 2025. The HBV‐positive patients were classified into three clinical subgroups based on the American Association for the Study of Liver Diseases (AASLD) 2017 guidelines: chronic hepatitis B (CHB, *n* = 102), LC (*n* = 96), and HCC (*n* = 231). LC was diagnosed based on a combination of clinical symptoms and characteristic biochemical abnormalities, including hyperbilirubinemia, elevated levels of aspartate aminotransferase (AST) and alanine aminotransferase (ALT), prolonged prothrombin time, and reduced serum albumin levels. Diagnosis was further supported by ultrasonographic evidence of chronic liver parenchymal damage. HCC was diagnosed using a combination of clinical assessment, imaging modalities (such as ultrasound or CT), and histopathological confirmation where available. Healthy controls were blood donors confirmed to be negative for hepatitis B surface antigen (HBsAg). All participants were negative for anti‐HCV and anti‐HIV antibodies, as confirmed by enzyme‐linked immunosorbent assay (ELISA) tests. None of the participants had a history of alcohol or drug abuse. Whole blood and serum samples were collected from each participant and stored at −80°C until further analysis.

### 2.3 SLC10A1 Genotyping

Human genomic DNA was extracted from whole blood using the QIAamp DNA Mini Kit (Qiagen, Hilden, Germany), according to the manufacturer's instructions. The DNA quality and quantity were assessed using the NanoPhotometer® P300 (Implen GmbH, Munich Germany). Exon 4 of the *SLC10A1* gene was amplified using the primers SLC10A1_E4 F (5′‐CCA TCG CTG CGA AAC TC‐3′) and SLC10A1_E4 R (5′‐GGG CTA CCT GGT TCT TAG TGA‐3′). PCR amplification was performed in 25 μL reaction volumes, including 1X Quick‐Load Taq Master Mix (New England Biolabs, Ipswich, MA, USA), 0.2 μM of each primer, and 50 ng of genomic DNA. The thermal cycling conditions were as follows: initial denaturation at 95°C for 30 s, followed by 35 cycles of denaturation at 95°C for 30 s, annealing at 55°C for 40 s, and extension at 68°C for 40 s, with a final extension at 68°C for 5 min. Amplicons were visualized on 1.5% agarose gels as a 406 bp band. PCR products were purified using ExoSAP‐IT PCR Product Cleanup (Thermo Fisher Scientific, Waltham, MA, USA), and 5 μL of purified products were used as templates for sequencing, performed with the BigDye Terminator v1.1 Cycle Sequencing Kit (Life Technologies GmbH, Darmstadt, Germany) on an ABI 3130xl DNA Sequencer (Applied Biosystems, Foster City, CA, USA). Genotypes of SNP rs2296651 were identified as wild type (CC), heterozygous (CT), and homozygous mutant (TT).

### 2.4 HBV Nested PCR and Sanger Sequencing

HBV DNA was extracted from plasma using the QIAamp DNA Mini Kit (Qiagen, Hilden, Germany), following the manufacturer's instructions. Nested PCR was performed to amplify the preS/S region (604 bp in length). The outer PCR used primers HBV‐preS/S‐outF (5′‐ATACTCTGTGGAAGGCTGGC‐3′) and HBV‐preS/S‐outR (5′‐TTGAGAGAAGTCCACCACGAG‐3′), while the inner PCR used primers HBV‐preS/S‐inF (5′‐TGTGGGTCACCATATTCTTGG‐3′) and HBV‐preS/S‐inR (5′‐TAACACGAGCAGGGGTCCTA‐3′). Amplification reactions were carried out in a final volume of 25 µL, containing 1× MM HotStartTaq Master Mix (Qiagen, Hilden, Germany), 0.2 μM of each forward and reverse primer, and the template (5 μL of amplicon for the outer reaction or 2 μL of outer PCR products for the inner reaction). The thermal cycling program for the outer PCR consisted of an initial denaturation at 95°C for 15 min, followed by 30 cycles of denaturation at 94°C for 30 s, annealing at 55°C for 30 s, and extension at 72°C for 60 s, with a final extension step at 72°C for 10 min. The inner PCR was performed under the same conditions, with an increased cycle number to 35 and an annealing temperature of 60°C. Amplicons were visualized by agarose gel electrophoresis. Positive samples were purified using the ExoSAP‐IT PCR Product Cleanup Reagent (Thermo Fisher Scientific, Waltham, MA, USA) and subsequently sanger sequenced using the BigDye™ Terminator v3.1 Cycle Sequencing Kit (Life Technologies GmbH, Darmstadt, Germany) on an Applied Biosystems 3130xl Genetic Analyzer (Applied Biosystems, Foster City, CA, USA).

### 2.5 Phylogenetic Analysis

The obtained sequences were trimmed in Seqman version 6.1 (DNASTAR, Lasergene, USA) and the resulting consensus sequence was obtained. Alignment was performed using MAFFT version 7.0 [13] using the G‐INS‐I model with 37 references representative of genotypes A‐J obtained from NCBI database (https://ncbi.nlm.nih.gov/projects/genotyping/). The phylogenetic tree was reconstructed using the neighbor‐joining method from the aligned sequences using MEGA 11 [14], employing the Maximum Likelihood method using the Kimura 2 parameter plus Gamma Distribution model (K2+G). The statistical robustness and reliability of the branching order were confirmed via bootstrapping with 1000 replicates. The resulting phylogenetic tree was annotated and visualized using the online tool iTOL v6 (https://itol.embl.de/) [15].

### 2.6 Quantitative Real‐Time PCR

A quantitative real‐time PCR was performed using the QuantiTect Multiplex PCR NoROX Kit (Qiagen GmbH, Hilden, Germany) on a LightCycler480‐II (Roche, Mannheim, Germany). Each real‐time PCR reaction was performed in 20 µL volume, consisting of 1× QuantiTect Multiplex PCR NoROX Master Mix, 0.5 μM of each forward primer HBV‐61 (5′‐GGACCCCTGCTCGTGTTACA‐3′) and reverse primer HBV‐62 (5′‐GAGAGAAGTCCACCACGAGTCTAGA‐3′), 0.25 μM of the probe HBV TM‐05 (5′‐FAM‐TGTTGACAARAATCCTCACAATACCRCAGA/3BHQ_1/‐3′), with 15‐20 ng (5 µL) of DNA. The cycling conditions included an initial denaturation at 95°C for 15 min, followed by followed by 45 cycles of 95°C for 10 s and 60°C for 60 s.

### 2.7 Statistical Analysis

Clinical parameters were expressed as median values with ranges for quantitative variables and absolute numbers and percentages for categorical data. Binary logistic regression models, adjusted for age and gender were used to assess the association between the NTCP S267F variant and HBV‐related liver diseases. Adjusted odds ratios (ORs) with 95% confidence intervals (CIs) were calculated. Chi‐square and Fisher's exact tests were used to examine differences in categorical variables, while Kruskal‐Wallis and Mann‐Whitney‐Wilcoxon tests were applied for comparison of quantitative variables. Statistical analyses were performed using SPSS version 22, with a significance threshold set at *p* < 0.05.

## 3 Results

### 3.1 Baseline Characteristics of Study Subjects

The demographic, laboratory and clinical characteristics of the 743 study participants are presented in Table 1. The healthy control (HC) group (*n* = 314) had a mean age of 32.5 years (range: 18–69) and consisted predominantly of males (76%). Among the 429 patients with HBV infection, the majority were male (84%) with a higher mean age of 58 years (range: 16–92). A significant increase in mean age was observed with advancing liver disease stages (*p* < 0.001). Significant differences were observed among HBV subgroups for several clinical parameters, including AST, ALT, AFP, total bilirubin, glucose, prothrombin and RBC count (all *p* < 0.05). In contrast, no statistically significant differences were observed in GGT, urea, creatinine, or WBC count across disease stages (*p* > 0.05; Table 1).

**Table 1:** Demographic and clinical characteristics of healthy controls and HBV patients.

Characteristics	HC (*n* = 314)	HBV patients (*n* = 429)	CHB (*n* = 102)	LC (*n* = 96)	HCC (*n* = 231)	*p* value
Age (years)	32.5 [18–69]	58 [16–92]	47 [20–76]	56 [21–85]	64 [25–92]	**< 0.001**
Gender M/F	239/75	360/69	72/30	78/18	210/21	n/a
AST (IU/L)	n/a	65 [3–2578]	127 [3–2578]	79 [26–1410]	56 [7–1986]	**0.001**
ALT (IU/L)	n/a	57 [2–3154]	167 [2–3154]	71 [5–2466]	51 [10‐1758]	**0.001**
GGT (IU/L)	n/a	118 [3–1137]	114 [3–780]	156 [14–906]	117 [14‐1137]	0.085
AFP (IU/L)	n/a	13 [0.5–10^5^]	3.6 [0.62–1660]	6 [1–1660]	44 [0.5‐10^5^]	**< 0.001**
Total bilirubin (µmol/L)	n/a	15 [2–509]	19.8 [2–431]	17 [5–509]	15 [6‐501]	**0.006**
Ure (mmol/l)	n/a	5 [0.4–55]	4.7 [0.4–54]	5.5 [2.2–55]	5.2 [1.8‐19.6]	0.075
Creatinin (umol/l)	n/a	72 [5–562]	70 [5–166]	72 [41–219]	74 [7–562]	0.111
Glucose (mmol/l)	n/a	5.5 [1.8–28.5]	5.4 [3.5–28.5]	5.5 [3.1–24.4]	5.7 [1.8–24.4]	**< 0.001**
Prothrombin (% of standard)	n/a	87 [11–126]	84 [23–126]	69 [14–116]	89 [11–122]	**< 0.001**
WBC (x10^9^/L)	n/a	6.9 [2.1–25]	6.8 [2.6–23.7]	6.6 [2.1–15]	7 [3–25]	0.18
RBC(x10^12^/L)	n/a	4.7 [1.9–7.8]	4.9 [3.2–6.6]	4.5 [1.9–7.3]	4.6 [2.3–7.8]	**< 0.001**
PLT (x10^9^/L)	n/a	174 [18–795]	198 [53–795]	145 [18–397]	181 [40–529]	**< 0.001**
HBV DNA (copies/ml)	n/a	6.2 × 10^4^ [100–10^9^]	3.3 × 10^4^ [110–10^9^]	5.0 × 10^5^ [140–10^9^]	2.3 × 10^4^ [100–6.5 × 10^8^]	**< 0.001**

*Note:* Values given are medians and ranges. Kruskal‐Wallis test was used to test differences of nonparametric data. *p*‐values less than 0.05 are highlighted in bold.

Abbreviations: AFP, alpha‐fetoprotein; AST and ALT, aspartate and alanine amino transferase; CHB, chronic hepatitis B; HC, healthy control; HCC, hepatocellular carcinoma; IU, international unit; LC, liver cirrhosis; n/a, not available; NR, normal range; PLT, platelets; RBC, red blood cells; WBC, white blood cells.

### 3.2 S267F Variant Associated With HBV Infection and Clinical Outcomes

Genotyping of the NTCP S267F variant was performed in 429 HBV patients and 314 HCs (Table 2). The heterozygous CT genotype was significantly less frequent in HBV patients (12%) compared to healthy controls (16%) (OR = 0.5, 95% CI: 0.3–0.9; adjusted *p* = 0.013) (Table 2), suggesting a potential protective role against HBV infection. Further subgroup analysis revealed that the CT genotype was significantly underrepresented in patients with chronic hepatitis B (CHB; 6%) and LC (6%) compared to HCs. The adjusted odds ratios indicated a 70% reduction in the risk of CHB (OR = 0.3, 95% CI: 0.1–0.8; *p* = 0.013) and a 60% reduction in the risk of LC (OR = 0.4, 95% CI: 0.1–0.9; *p* = 0.041) (Table 2). However, no significant difference in the CT genotype frequency was observed between HCC patients (17%) and HCs (OR = 0.9, 95% CI: 0.5–1.8; adjusted *p* = 0.8) (Table 2), suggesting that the protective effect of the variant does not extend to HCC development.

**Table 2:** Association of NTCP variant (rs2296651) with HBV‐related liver diseases.

NTCP rs2296651	HC *n* = 314 (%)	HBV *n* = 429 (%)	CHB *n* = 102 (%)	LC *n* = 96 (%)	HCC *n* = 231 (%)	HBV versus HC		CHB versus HC		LC versus HC		HCC versus HC	
						OR (95%CI)	*p* value	OR (95%CI)	*p*‐value	OR (95%CI)	*p*‐value	OR (95%CI)	*p* value
CC	254 (84%)	378 (88%)	96 (94%)	90 (94%)	192 (83%)	Reference	**0.013**	Reference	**0.013**	Reference	0.041	Reference	0.8
CT	60 (16%)	51 (12%)	6 (6%)	6 (6%)	39 (17%)	0.5 (0.3‐0.9)		0.3 (0.1–0.8)		0.4 (0.1–0.9)		0.9 (0.5–1.8)	

*Note: p*‐values were calculated using binary logistic regression model adjusted for age and gender. *p*‐values less than 0.05 are highlighted in bold.

Abbreviations: CHB, chronic hepatitis B; HC, healthy controls; HCC, hepatocellular carcinoma; LC, liver cirrhosis; n, numbers genotyped; OR, odd ratio.

### 3.3 S267F Variant on Clinical Parameters in HBV Patients

**Figure 1:**
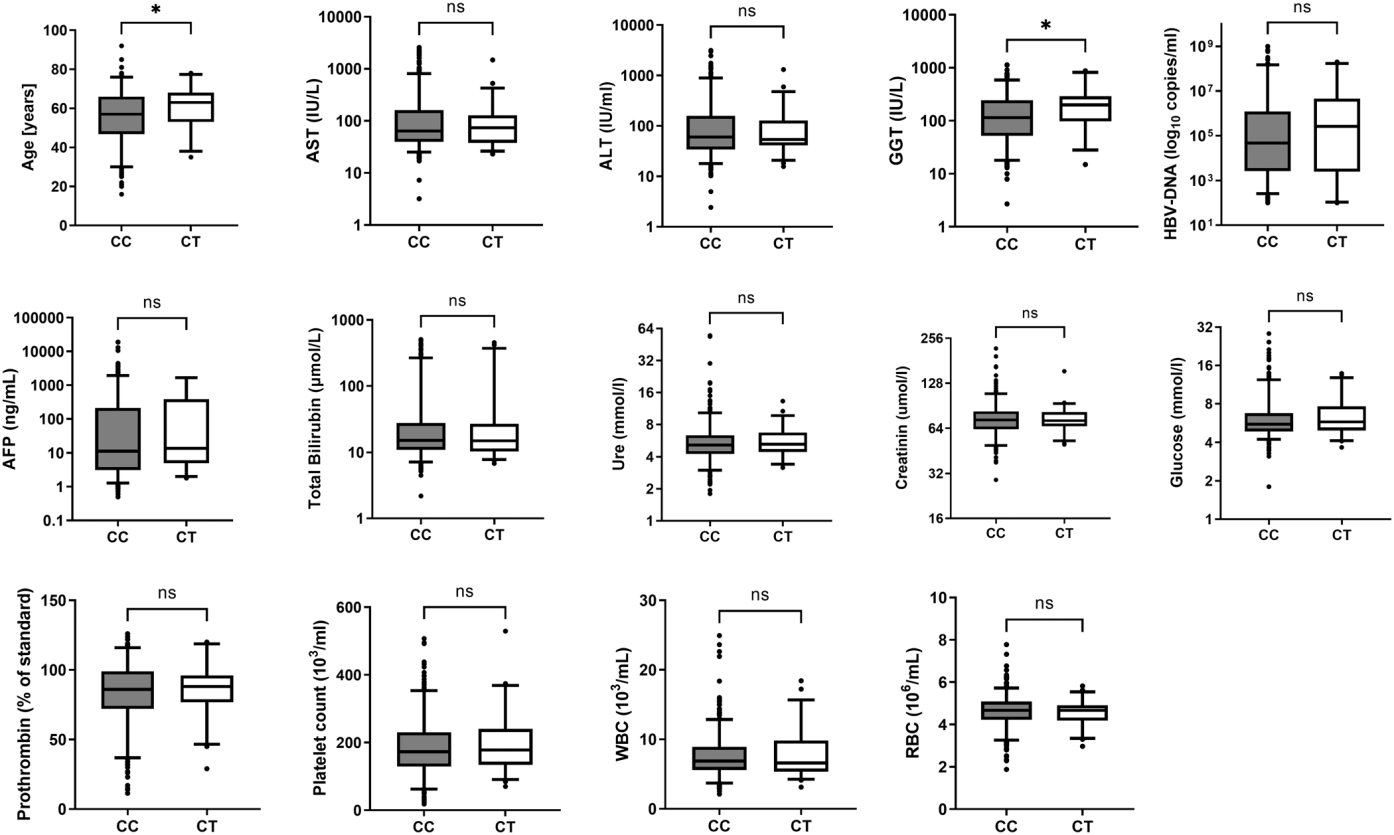
Association of the NTCP S267F variant with various biochemical parameters and viral load in HBV infected individuals. Laboratory parameters were compared between patients with the CC genotype and patients with the CT genotype. Boxplots illustrate medians with interquartile ranges. Adjusted *p*‐values were calculated using the Mann‐Whitney‐Wilcoxon test.

As shown in Figure 1, after adjusting for age and sex, the CT genotype was significantly associated with lower age and altered GGT levels (*p* < 0.05). However, no significant associations were found between genotype and other clinical or biochemical parameters, including AST, ALT, HBV DNA levels, AFP, total bilirubin, urea, creatinine, glucose, prothrombin time, platelet count, or WBC and RBC counts (all *p* > 0.05). These findings suggest that while the S267F variant may influence some aspects of HBV‐related pathology, it does not broadly impact most clinical parameters.

### 3.4 Association Between HBV Genotypes, Disease Progression, and NTCP Variants

**Figure 2:**
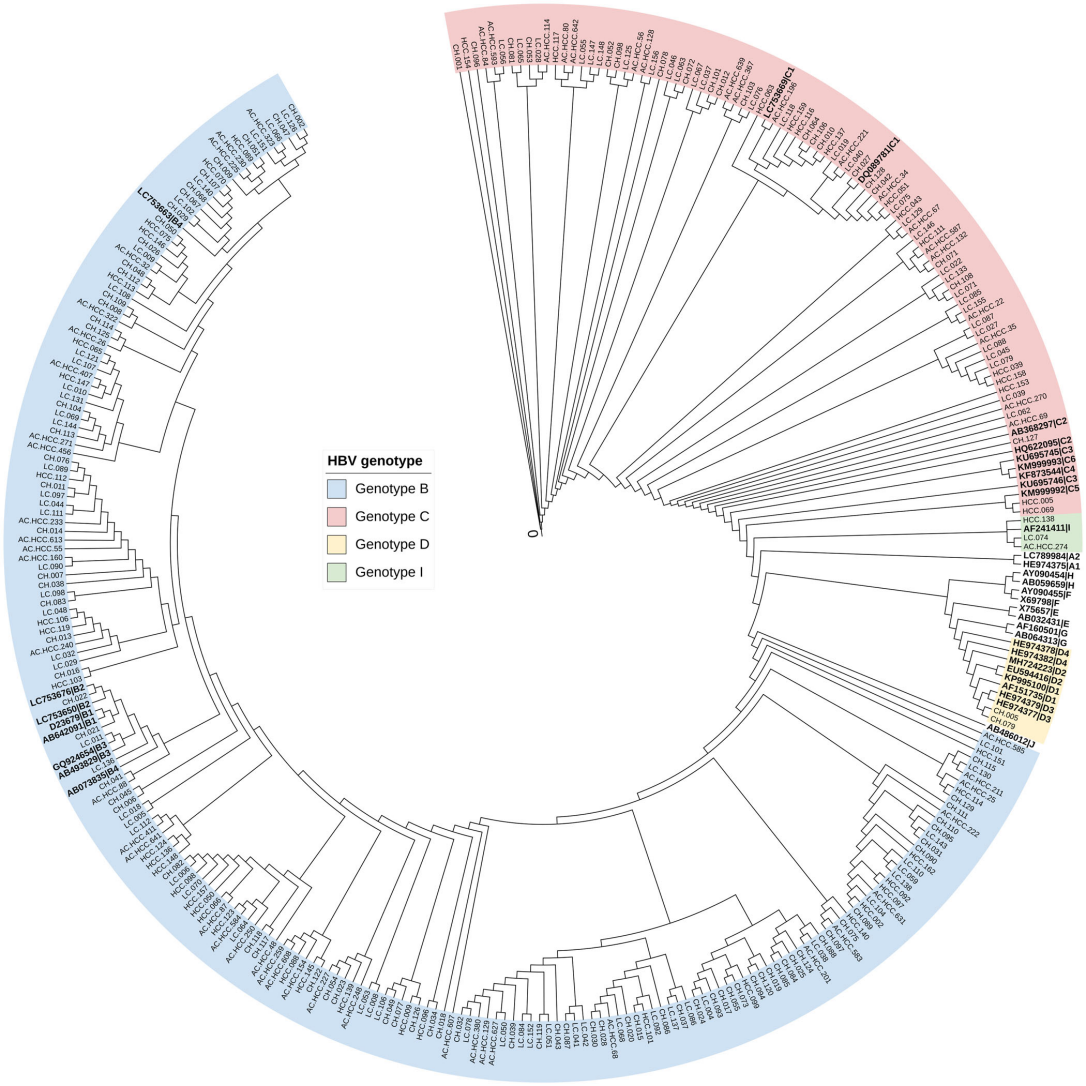
Phylogenetic tree of the HBV PreS/S gene constructed using the Neighbor‐joining method. The tree was generated using the Kimura 2‐parameter model with a gamma distribution (K2+G), based on 273 HBV sequences obtained in this study and 37 reference nucleotide sequences representing HBV genotypes A–J (highlighted in bold). HBV sequences from this study clustered within genotypes B, C, D, and I.

HBV genotyping was successful in 273/314 patients. Phylogenetic analysis (Figure 2) showed genotype B as the most prevalent (70%), followed by genotype C (28%). Genotypes D and I were infrequently observed (0.7% and 1.1%, respectively). No significant association was found between the S267F genotype and HBV genotype distribution across CHB, LC, or HCC subgroups (Table 3; *p* > 0.05). However, when comparing clinical outcomes by genotype, infection with genotype C was associated with significantly increased risk of LC (OR = 2.4, 95% CI: 1.2–5.0; *p* = 0.015) and HCC (OR = 2.5, 95% CI: 1.0–5.8; *p* = 0.039) compared to genotype B (Table 4), indicating a stronger pathogenic potential of genotype C.

**Table 3:** NTCP variant (rs2296651) and distribution of different HBV genotypes on HBV related liver diseases.

Category		NTCP variant		Total	*p* value*
		CC	CT		
CHB (*n* = 98)	genotype B	69	6	75	*p* > 0.05
	genotype C	21	0	21	
	genotype D	2	0	2	
LC (*n* = 85)	genotype B	48	5	53	*p* > 0.05
	genotype C	30	1	31	
	genotype I	1	0	1	
HCC (*n* = 90)	genotype B	54	9	63	*p* > 0.05
	genotype C	20	5	25	
	genotype I	2	0	2	

Abbreviations: CHB, chronic hepatitis B; HCC, hepatocellular carcinoma; LC, liver cirrhosis.

* *p*‐values were calculated using Chi‐square test.

**Table 4:** Association of HBV genotype with the risk of liver cirrhosis (LC) and hepatocellular carcinoma (HCC).

HBV genotype	CHB versus LC		CHB versus HCC	
	OR (95% CI)	*p* value	OR (95% CI)	*p* value
Genotype B	Reference	**0.015**	Reference	**0.039**
Genotype C	2.4 (1.2–5.0)		2.5 (1.0–5.8)	

## 4 Discussion

The sodium taurocholate cotransporting polypeptide plays a dual role as a hepatic bile acid transporter and a critical entry receptor for both HBV and HDV [8, 16]. The S267F polymorphism (rs2296651) within NTCP is a well‐characterized functional variant that impairs viral entry into hepatocytes by altering the receptor's conformation. This substitution (serine to phenylalanine) has been shown to reduce susceptibility to HBV infection and progression to severe liver disease in East Asian populations [9, 17, 18]. In our cohort of Vietnamese individuals, the heterozygous CT genotype of S267F was present in 16% of healthy controls, a relatively high frequency compared to other Asian populations such as China, Korea, and Japan [9, 19, 20, 21]. Importantly, we observed a significantly lower frequency of the CT genotype among HBV‐infected patients, particularly those with CHB and LC, where the prevalence dropped to approximately 6%. These associations translate to a twofold to threefold reduced risk for CHB and LC, reinforcing the protective role of the S267F variant and findings consistent with previous studies [9, 18, 21].

Mechanistically, recent structural studies indicate that S267 lies within a key a critical surface interface of NTCP required for binding the HBV‐preS1 peptide in its outward‐facing conformation. The S267F mutation introduces a bulky, hydrophobic phenylalanine, which likely causes steric hindrance and disrupts the local architecture of the viral binding site. This substitution may also interfere with adjacent sodium‐binding sites, thereby restricting NTCP's conformational flexibility and promoting an inward‐facing, closed conformation that is incompatible with HBV attachment and entry. These structural alterations align with functional evidence showing markedly reduced HBV infectivity and impaired bile acid transport in cells expressing the S267F variant. Together, these effects may contribute to the partial protection against HBV infection and the reduced risk of liver disease progression observed in carriers of this mutation [8, 22, 23]. However, our findings diverge from some earlier reports regarding HCC. Unlike previous studies that observed reduced S267F frequencies among HCC patients [10], we found no significant difference in the CT genotype between HCC cases (17%) and healthy controls (16%). This discrepancy may reflect population‐specific dynamics, limited statistical power, or complex HCC pathogenesis that extends beyond viral entry mechanisms.

We also explored the impact of the S267F variant on clinical and laboratory parameters. Aside from higher GGT levels in CT carriers, there were no significant differences across other markers, including HBV DNA load. This suggests that while the S267F variant may impede viral entry, it does not necessarily affect viral replication or systemic viral load. Our results align with the hypothesis that NTCP polymorphisms modulate initial susceptibility more than ongoing viral kinetics.

Regarding viral genotypes, our study reaffirmed genotype B (70%) as the predominant HBV strain in Vietnam, followed by genotype C (28%) [24, 25], with minor representation of genotypes D and I (2%). These distributions are consistent with earlier reports from Vietnam [24, 26]. Notably, genotype C was associated with a 2.5‐fold increased risk of progression to cirrhosis and HCC compared to genotype B, echoing the known higher virulence and prolonged replication phase of genotype C [27]. We further examined whether the protective effect of the S267F variant was modulated by HBV genotype. No significant differences in CT genotype distribution were observed between genotypes B and C within disease subgroups (CHB, LC, HCC), suggesting that the variant's protective effect is likely genotype independent. However, given the limited number of CT carriers within each genotype‐disease subgroup, future studies with larger sample sizes are warranted to validate this observation. Interestingly, our phylogenetic analysis identified rare occurrences of genotypes D and I. Genotype D, common in the Mediterranean, is generally associated with less aggressive disease [28, 29], while genotype I, an X/C recombinant recently reported in Vietnam and Laos, requires further characterization through whole‐genome sequencing [30].

This study supports a protective role for the NTCP S267F variant in HBV infection and cirrhosis in the Vietnamese population. However, several limitations should be acknowledged. First, we did not assess mutations in the HBV PreS1 region, which directly interacts with NTCP and may influence viral entry. Second, subgenotype‐level analyses and integration of whole‐genome viral sequencing could provide deeper insights into host‐virus interactions. Finally, our cross‐sectional design limits causal inference.

Our findings reinforce the protective association of the NTCP S267F variant against HBV infection and progression to cirrhosis but not against HCC. The effect appears to be independent of HBV genotype, although further research is needed to explore genotype‐specific interactions. These results underscore the importance of host genetic variation in shaping HBV clinical outcomes and may inform future risk stratification and therapeutic strategies in HBV‐endemic regions.

## Author Contributions

Thirumalaisamy P. Velavan, Nguyen Linh Toan, Le Huu Song designed, supervised the study and contributed to the study materials for all experimental investigations. Pham Xuan Huy, Le Chi Cao, Dao Thi Huyen, Tran Thi Thu Hien, Truong Nhat My, Tran Thi Thanh Huyen participated in the study design. Pham Xuan Huy, Dao Thi Huyen, Tran Thi Thu Hien, Truong Nhat My, Tran Thi Thanh Huyen collected samples. Pham Xuan Huy, Dao Thi Huyen, Truong Nhat My performed experimental procedures. Pham Xuan Huy, Le Chi Cao performed statistical and phylogenetic analysis. Pham Xuan Huy, Le Chi Cao wrote the first draft. Thirumalaisamy P. Velavan, Nguyen Linh Toan revised the first draft. All authors have read and approved the manuscript.

## Conflicts of Interest

The authors declare no conflicts of interest.

## Data Availability

Data sharing is not applicable to this article as no new data were created or analyzed in this study.
